# High-Throughput Screening Platform To Identify Inhibitors of Protein Synthesis with Potential for the Treatment of Malaria

**DOI:** 10.1128/aac.00237-22

**Published:** 2022-06-01

**Authors:** Fabio Tamaki, Fabio Fisher, Rachel Milne, Fernando Sánchez-Román Terán, Natalie Wiedemar, Karolina Wrobel, Darren Edwards, Hella Baumann, Ian H. Gilbert, Beatriz Baragana, Jake Baum, Susan Wyllie

**Affiliations:** a Wellcome Centre for Anti-Infectives Research, School of Life Sciences, University of Dundee, Dundee, United Kingdom; b Department of Life Sciences, Imperial College London, London, United Kingdom; c School of Medical Sciences, University of New South Wales, New South Wales, Sydney, Australia

**Keywords:** *Plasmodium*, malaria, *in vitro* translation, protein synthesis, drug discovery, antimalarials

## Abstract

Artemisinin-based combination therapies have been crucial in driving down the global burden of malaria, the world’s largest parasitic killer. However, their efficacy is now threatened by the emergence of resistance in Southeast Asia and sub-Saharan Africa. Thus, there is a pressing need to develop new antimalarials with diverse mechanisms of action. One area of *Plasmodium* metabolism that has recently proven rich in exploitable antimalarial targets is protein synthesis, with a compound targeting elongation factor 2 now in clinical development and inhibitors of several aminoacyl-tRNA synthetases in lead optimization. Given the promise of these components of translation as viable drug targets, we rationalized that an assay containing all functional components of translation would be a valuable tool for antimalarial screening and drug discovery. Here, we report the development and validation of an assay platform that enables specific inhibitors of Plasmodium falciparum translation (*Pf*IVT) to be identified. The primary assay in this platform monitors the translation of a luciferase reporter in a P. falciparum lysate-based expression system. Hits identified in this primary assay are assessed in a counterscreen assay that enables false positives that directly interfere with the luciferase to be triaged. The remaining hit compounds are then assessed in an equivalent human IVT assay. This platform of assays was used to screen MMV’s Pandemic and Pathogen Box libraries, identifying several selective inhibitors of protein synthesis. We believe this new high-throughput screening platform has the potential to greatly expedite the discovery of antimalarials that act via this highly desirable mechanism of action.

## INTRODUCTION

Malaria is a life-threatening disease that results in more than 400,000 deaths every year, many of which occur among children under the age of 5 years (1). The disease results from infection with unicellular, protozoan parasites from the genus *Plasmodium*, with the vast majority of deaths caused by Plasmodium falciparum and P. vivax. Current front-line therapies for malaria are under constant threat from the emergence of drug resistance (2, 3). The current standard of care for the treatment of malaria, recommended by the World Health Organization (WHO), is reliant upon artemisinin-based combination therapies (ACTs) (4). However, clinical artemisinin resistance is now prevalent across Southeast Asia (5) and sub-Saharan Africa (6), threatening these combination therapies. Thus, there is a pressing need for new and effective drugs to provide chemoprotection, prevent transmission, and treat (*vivax*) relapse.

The development of new drugs capable of treating this devastating parasitic disease has been confounded by a number of factors. The life cycle of the *Plasmodium* parasite is extremely complex. Following initial transmission via the bite of the *Anopheles* mosquito, sporozoites infect the hepatocytes of the host liver. Parasites replicate and differentiate within hepatocytes prior to entering the bloodstream, where merozoites infect red blood cells. Intraerythrocytic infection is characterized by a rapid expansion of the parasite population (schizogony). At this stage, some parasites differentiate into sexual forms (gametocytes) that can be taken up through the bite of a mosquito and transmitted to other humans. Long-term control of malaria will likely require the development of multiple compounds that demonstrate activity against multiple parasite life cycle stages, extremely challenging in terms of drug discovery. In addition, antimalarial drug discovery has been hindered by the general lack of robustly validated drug targets in *Plasmodium*, severely limiting target-focused screening programs.

One area of *Plasmodium* metabolism that has proven relatively rich in exploitable antimalarial targets is protein synthesis. Doxycycline is widely used for malaria chemoprophylaxis and assumed to act through inhibition of parasite protein synthesis via binding to the 30S ribosomal subunit. The fungal secondary metabolite cladosporin, a potent inhibitor of *Plasmodium* growth in blood and liver stages, specifically targets lysyl-tRNA synthetase (LysRS) (7). Optimization of a chromone hit identified in a biochemical screen led to the first P. falciparum LysRS (*Pf*LysRS) inhibitor with efficacy in a malaria mouse model (8). In addition to LysRS, a series of novel bicyclic azetidines demonstrating *in vivo* efficacy were found to specifically target cytosolic phenylalanyl-tRNA synthetase (PheRS) (9), while the potent antimalarials borrelidin and halofuginone inhibit threonyl-tRNA synthetase (ThrRS) (10) and prolyl-tRNA synthetase (ProRS) (11), respectively. Aminoacyl-tRNA synthetases catalyze aminoacylation of tRNAs with their cognate amino acids. P. falciparum translation elongation factor 2 (*Pf*eEF2), responsible for the GTP-dependent translocation of the ribosome along mRNA, has also been identified as a promising target (12). Indeed, M5717, a compound specifically targeting *Pf*eEF2, is now undergoing first-in-human trials (13).

The advantage of antimalarials that target core, essential biological processes, such as protein synthesis, is that they have the potential to be effective against multiple life cycle stages of *Plasmodium*. Strategies that can facilitate the identification of protein synthesis inhibitors with diverse modes of action are highly desirable. Previous studies have reported the development of cell-free or *in vitro* translation (IVT) assays capable of identifying antimalarials that target protein synthesis (14–16). Their use has enabled the screening of small libraries as well as validation of the drug mechanism of action (MoA) with respect to protein translation (15). However, these assays have not yet proven suitable or scalable to support high-throughput screening of larger compound libraries. Here, we describe the development of an assay platform devoted to the identification of specific inhibitors of P. falciparum translation. This platform is comprised of a P. falciparum lysate-based IVT assay that monitors the translation of a luciferase reporter, a counterscreen assay to identify false positives that interfere directly with the luciferase reporter, and an equivalent human IVT assay that is used to guide the identification of selective inhibitors of parasite translation. These 384-well plate assays are capable of identifying a diverse range of translation-specific inhibitors. To demonstrate the power and utility of this platform, pilot screens of the Medicines for Malaria Venture (MMV) open-access Pathogen Box and Pandemic Box compound libraries were carried out, leading to the identification of several novel inhibitors of P. falciparum protein synthesis.

## RESULTS AND DISCUSSION

### Optimization and validation of high-throughput *Pf*IVT and *Hs*IVT assays.

**FIG 1:**
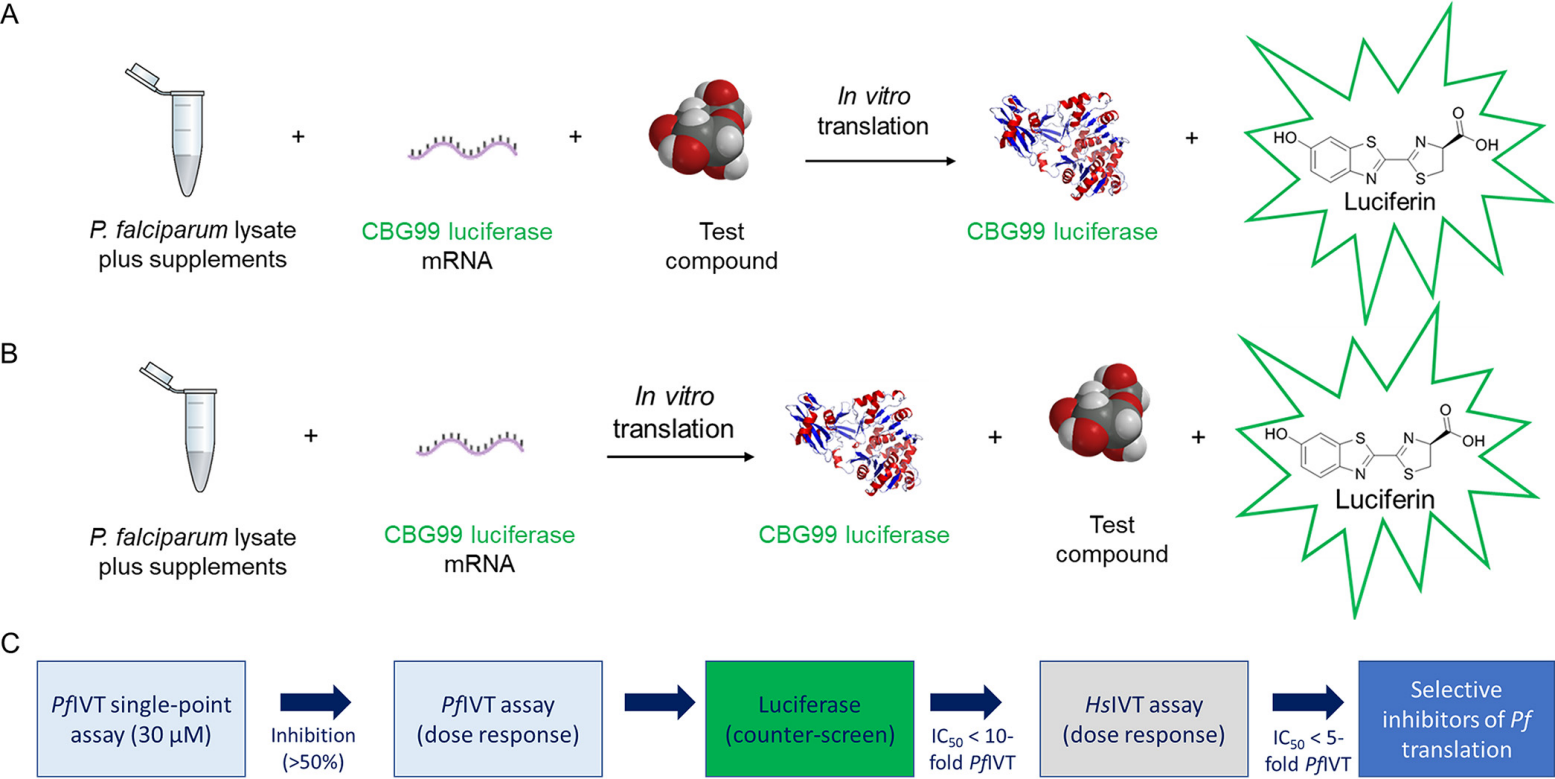
(A and B) Schematic representation of *Pf*IVT (A) and luciferase counterscreen (B) assays. (C) Assay workflow and criteria for progression.

Our P. falciparum *in vitro* translation (*Pf*IVT) assay is built upon previously established assays (14, 17) and is summarized in Fig. 1A. In this assay, successful reconstitution of *Plasmodium* protein translation is reported via synthesis of a luciferase reporter. For *Pf*IVT, two independent untranslated regions (UTRs), previously shown to promote strong levels of translation (17), were introduced upstream and downstream of the luciferase. The 5′ UTR from P. falciparum histidine-rich protein 3 (*Pf*Hrp3) (18) was introduced upstream of the reporter gene, and the 3′ UTR from P. falciparum histidine-rich protein 2 (*Pf*Hrp2) gene (19) was introduced downstream. For the reporter construct to support our complementary human IVT assay (*Hs*IVT), the 5′ UTR was replaced with an internal ribosome entry site (IRES) from encephalomyocarditis virus (EMCV) (20) and the 3′ UTR was replaced with a poly(A) tail (21). It is worth noting that the 5′ UTR of EMCV was unable to initiate translation in the *Pf*IVT assay and vice versa. Finally, a 30-bp poly(A) tail, present within the vector and downstream of the luciferase gene, was inserted to enhance mRNA stability (21).

Translationally active P. falciparum or human embryonic kidney (HEK) 293F lysates were supplemented with accessory, helper, amino acid, and energy regeneration solutions (see Materials and Methods for details). *In vitro* translation reactions were initiated by the addition of purified mRNA to each reaction well. Using this basic assay format, a number of parameters were assessed and optimized, namely, assay temperature, time, mRNA concentration, and reaction volume (summarized in Fig. S2 in the supplemental material). In addition, we compared the luminescence signal of the CBG firefly luciferase with CBG99, derived from the click beetle Photinus pyralis (18). Direct comparison of these two luciferases revealed that the luminescence signal for CBG99 was over 2-fold higher than for the original firefly luciferase (Fig. S2), leading us to base our reporter constructs around this superior luciferase. Based on these preliminary studies, the optimal assay parameters were established as reaction volume of 5 μL, assay temperature of 32°C, mRNA concentration of 1,000 ng/μL, and reaction time of 210 min. Under these conditions, the assay reported a signal-to-background ratio of at least 10 and robust Z′ values above 0.5 in a 384-well plate format.

**FIG 2:**
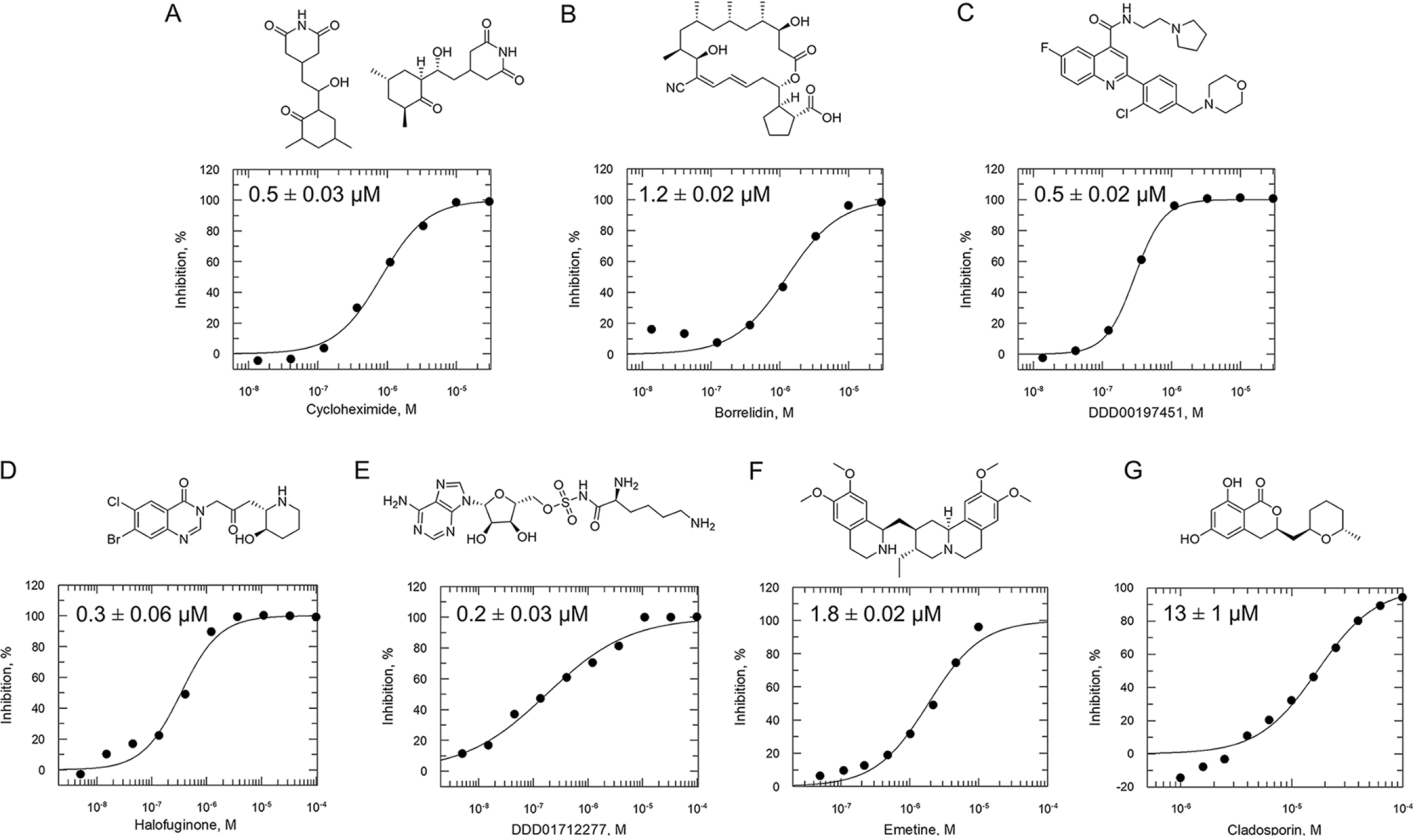
Established inhibitors of translation. Compounds that are known to inhibit different aspects of *in vitro* translation were selected to validate our IVT assay. (A) Cycloheximide is an inhibitor of the translocation step in elongation (46). (B) Borrelidin is an inhibitor of ThrRS (47). (C) DDD00197451 is an inhibitor of translation via eEF2 (44). (D) Halofuginone is an inhibitor of ProRS (11). (E) DDD001712277 is an inhibitor of LysRS. (F) Emetine is an inhibitor of the 80S ribosome (43). (G) Cladosporin is an inhibitor of LysRS (7). All curves shown are from a single technical replicate and are representative of data for at least two biological replicates. IC_50_ values (insets) are weighted means ± SD from at least two biological replicates.

A selection of established inhibitors of protein translation in *Plasmodium* was then used to validate the *Pf*IVT assay, including cycloheximide (inhibitor of the translocation step in the elongation), emetine (inhibitor of 80S ribosome), borrelidin (inhibitor of ThrRS), halofuginone (inhibitor of ProRS), cladosporin (inhibitor of LysRS), and an analogue of DDD107498/M5717 (inhibitor of eEF2), now in clinical trials for the treatment of malaria (Fig. 2). In addition, an inhibitor that mimics a transition state analogue of LysRS (DDD01712277) was also assessed. These compounds, known to inhibit various aspects of cytosolic protein translation in *Plasmodium*, were successfully identified by this assay, validating its ability to identify known translation inhibitors (50% inhibitory concentration [IC_50_] values summarized in Fig. 2). In contrast, doxycycline and clindamycin, reported to target protein translation within the apicoplast of P. falciparum (22, 23), were not active against *Pf*IVT at 100 μM. Collectively, these data illustrate the utility of the *Pf*IVT assay to identify inhibitors of cytoplasmic translation in P. falciparum with a broad range of different mechanisms of action. Cycloheximide, emetine, and halofuginone were also used to validate the *Hs*IVT assay and, as expected, were found to successfully inhibit translation in this assay format (Fig. S3), while the P. falciparum-specific inhibitor cladosporin was inactive in this assay at 100 μM.

### Luciferase counterscreen.

To confirm that actives identified in IVT assays are bona fide inhibitors of translation, rather than false positives interfering with the CBG99 luciferase reporter, a counterscreen was established (Fig. 1B). This assay enables the direct inhibition of CBG99 luciferase to be monitored and was validated using luciferase inhibitor I. Using luciferase expressed in IVT reactions, the established luciferase inhibitor I reported an IC_50_ value of 5.5 μM (Fig. S4). Subsequently, false positives were identified as compounds with IC_50_ values less than 1 order of magnitude (10-fold) higher for the *Pf*IVT assay than for the luciferase counterscreen.

### Screening of MMV’s Pathogen and Pandemic Boxes.

The Pathogen Box (https://www.mmv.org/mmv-open/pathogen-box) and the Pandemic Box (https://www.mmv.org/mmv-open/pandemic-response-box) are collections of 400 diverse, drug-like compounds that have previously demonstrated some level of activity against neglected tropical diseases (NTDs), bacteria, viruses, or fungi. MMV provide these small libraries free of charge to stimulate much-needed drug discovery programs and to take advantage of potential pathogen-hopping opportunities.

**FIG 3:**
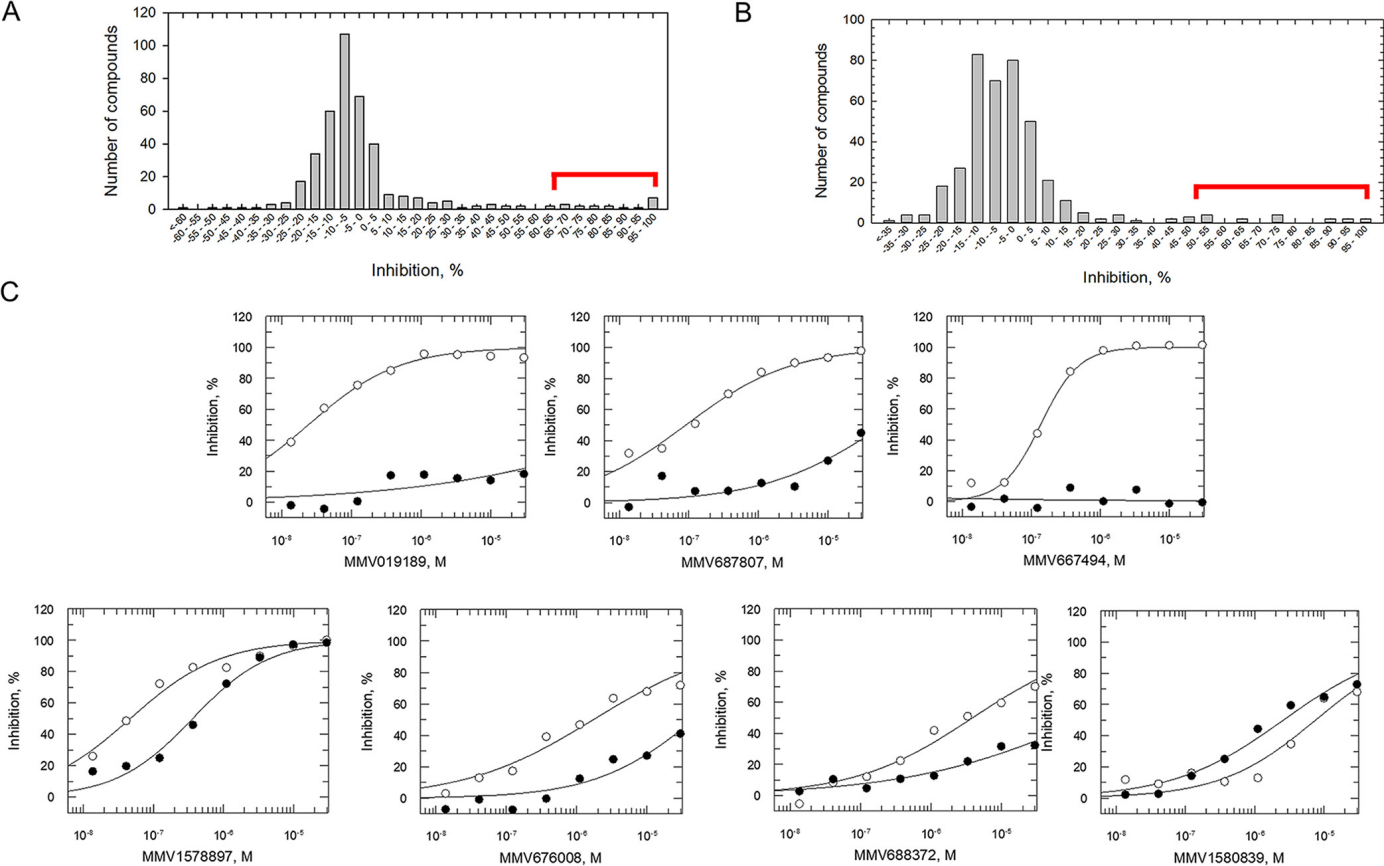
Assessment of the Pathogen Box and the Pandemic Box open access compound libraries against P. falciparum IVT assay. (A) Single-point (30 μM) high-throughput screen of the 400 compounds contained within the Pathogen Box against the IVT assay. Compounds demonstrating >58.02% inhibition were identified as hits (18 compounds; 4.5% hit rate) (highlighted in red). Mean Z′ = 0.855. (B) Single-point (30 μM) screen of the Pandemic Box (400 compounds) against *Pf*IVT assay. Compounds demonstrating >47.29% inhibition were identified as hits (14 compounds; 3.5% hit rate) (highlighted in red). The mean Z′ of the assay was 0.825. (C) Assessment of selected hit compounds in 8-point potency assays (closed circles) and a luciferase counterscreen (open circles). All curves shown are from a single technical replicate but representative of data from two biological replicates. IC_50_ values are summarized in Tables 1 and 2.

Both compound libraries were submitted for assessment in our IVT assay platform (Fig. 1C). Initially, compounds were screened in single point (30 μM) against the *Pf*IVT assay. Analysis of our single-point screens revealed normal distributions of inhibition (for the Pathogen Box, the median inhibition [x̃] was 1.00% and the standard deviation [σ] was 23.61% [Fig. 3A]; for the Pandemic Box, x̃ was 2.08% and σ was 19.75% [Fig. 3B]). DDD00197451, an analogue of eEF2 inhibitor M5717, was added as an internal control in all plates and inhibited >97% translation (*Pf*IVT), comparable to the positive control cycloheximide. Interestingly, both libraries presented nitazoxanide (MMV688991) among the primary hits, supporting the robustness of *Pf*IVT assay in identifying hits independently. Using a cutoff of 2.5σ (98.7% confidence assuming a normal hit distribution), *Pf*IVT assay identified hit rates of 4.5% for the Pathogen Box (18 compounds demonstrating >58.02% inhibition) and 3.5% for the Pandemic Box (14 compounds demonstrating >47.29% inhibition). These represent unusually high hit rates compared to standard target-based screens but were consistent with hit rates seen for high-content and cell-based screens (24). Our interpretation is that since translation is a complex, multicomponent process offering multiple potential targets, the *Pf*IVT assay aligns more closely with phenotype-based screens. It should also be noted that the Pathogen Box contains a number of compounds confirmed to inhibit P. falciparum growth *in vitro* (25), which could have positively influenced the hit rate seen in this study.

Primary hits identified in the single-point screen were reassessed in dose-dependent *Pf*IVT assays to confirm their activity and accurately measure their potency. Six primary hits from the Pathogen Box (out of 18) and 4 from the Pandemic Box (out of 14) presented IC_50_ values of <1 μM, with the most potent (nitazoxanide [MMV688991]) returning an IC_50_ value of 20 nM (Table 1). To confirm hits as bona fide inhibitors of *in vitro* translation, rather than false positives interfering with the CBG99 luciferase reporter, compounds were next assessed against our validated luciferase counterscreen. False positives were identified as compounds presenting IC_50_ values <10-fold more potent in the *Pf*IVT assay than in the counterscreen. Using this criterion, two compounds from the Pathogen Box were excluded as false positives: the most potent hit, MMV688991/nitazoxanide, a broad-spectrum anti-infective (26, 27), as well as MMV687243. Thus, 16 compounds from the Pathogen Box were confirmed as inhibitors of *Pf*IVT, with 5 compounds demonstrating submicromolar potency (Table 1). Remarkably, 10 of the 12 hit compounds from the Pandemic Box demonstrated similar IC_50_ values in the *Pf*IVT and the counterscreen (Table 2), indicating that these are inhibitors of the CBG99 luciferase rather than translation. The remaining two confirmed *Pf*IVT inhibitors (MMV1578897 and MMV1580839) both demonstrated submicromolar potency, with MMV1578897 particularly active (IC_50_ value, 40 nM).

**TABLE 1:** Collated *Pf*IVT and counterscreen data for compounds from MMV’s Pathogen Box demonstrating >50% inhibition in a single-point IVT screen at 30 μM^*a*^

Compound	*Pf*IVT inhibition at 30 μM (%)	IC_50_ value (μM)		
		*Pf*IVT	*Hs*IVT	Counterscreen
MMV688407	98.7	6 ± 0.09	5.2 ± 0.02	>30
MMV667494	97.7	0.3 ± 0.09	>30	>30
MMV688991	97.0	0.02 ± 0.008	0.04 ± 0.0003	0.02 ± 0.008
MMV688547	96.7	1.8 ± 0.07	2.7 ± 0.02	>30
MMV687807	95.1	0.09 ± 0.007	0.5 ± 0.006	25 ± 0.8
MMV688362	93.5	2.8 ± 0.08	5.6 ± 0.04	>30
MMV634140	88.4	2.9 ± 0.09	>30	>30
MMV019189	84.4	0.03 ± 0.009	7.7 ± 0.3	7 ± 0.6, (>30)
MMV687243	82.5	2.2 ± 0.08	13 ± 0.2	0.9 ± 0.09
MMV637953	78.7	3.1 ± 0.07	>30	>30
MMV688474	78.4	16 ± 0.9	14 ± 0.07	>30
MMV676008	74.9	0.4 ± 0.08	12 ± 0.4	21 ± 0.6
MMV687730	72.4	3.9 ± 0.8	>30	>30
MMV675998	68.9	13 ± 0.9	18 ± 0.13	>30
MMV688271	65.7	18 ± 0.9	13 ± 0.16	>30
MMV676350	65.1	16 ± 0.8	6.7 ± 0.13	>30
MMV687188	64.9	2.4 ± 0.07 (>30)	>30	>30
MMV688372	62.9	0.6 ± 0.06	>30	11 ± 0.8 (>30)
MMV676512	54.2	>30	22 ± 0.46	>30

a All IC_50_ values represent the weighted means ± standard deviations of two technical replicates. Compounds with IC_50_ values 1 order of magnitude (10-fold) higher for the counterscreen than for the *Pf*IVT assay are considered viable hits and are shaded.

**TABLE 2:** Collated *Pf*IVT and counterscreen data for compounds from MMV’s Pandemic Box demonstrating >50% inhibition in a single-point IVT screen at 30 μM^*a*^

Compound	*Pf*IVT inhibition at 30 μM (%)	IC_50_ value (μM)		
		*Pf*IVT	*Hs*IVT	Counterscreen
MMV688991	98.3	0.02 ± 0.007	0.04 ± 0.0003	0.04 ± 0.008
MMV002459	87.3	29 ± 0.9 (>30)	22 ± 0.3	>30
MMV1578897	93.4	0.04 ± 0.008	0.18 ± 0.001	0.4 ± 0.08
MMV1582495	89.9	0.6 ± 0.07	3.1 ± 0.04	1.7 ± 0.08
MMV1578578	74.6	3.7 ± 0.07	10 ± 0.12	1.3 ± 0.08
MMV1579781	72.4	8.1 ± 0.09	>30	0.5 ± 0.08
MMV1580839	73.3	0.8 ± 0.08	7.6 ± 0.12	8.3 ± 0.08
MMV124656	72.6	14 ± 0.9	17 ± 0.16	3.8 ± 0.08
MMV108465	62.6	17 ± 0.8	23 ± 0.11	7.6 ± 0.5
MMV247764	63.2	14 ± 0.7	23 ± 0.2	3.2 ± 0.06
MMV141011	51.8	>30	>30	10 ± 0.8
MMV003291	52.9	2 ± 0.07	>30	0.5 ± 0.08
MMV1634391	52.4	17 ± 0.6	1.1 ± 0.01	>30

a All IC_50_ values represent the weighted mean ± standard deviation of two technical replicates. Compounds with IC_50_ values 1 order of magnitude (10-fold) higher for the counterscreen compared to the *Pf*IVT assay are considered viable hits and are shaded.

The 18 confirmed inhibitors of P. falciparum translation were assessed in *Hs*IVT assays to detect potential liabilities as inhibitors of human translation. As expected, MMV667494, an analogue of the *Pf*eEF2 inhibitor M5717, was inactive against *Hs*IVT (IC_50_ value, >30 μM), in keeping with compounds from this series previously demonstrating a high degree of selective inhibition for P. falciparum growth compared to human cells (12). In contrast, the selectivity index for compound MMV687807 inhibiting *Pf*IVT (IC_50_, 0.09 μM) compared to *Hs*IVT (IC_50_, 0.5 μM) was ≤5-fold, earmarking this compound as potentially a generic inhibitor of translation. Indeed, the established ribosome inhibitor emetine (*Hs*IVT IC_50_, 1.4 μM; *Pf*IVT IC_50_, 0.35 μM) demonstrated a similarly narrow selectivity window. Combined, these data demonstrate the importance of our *Hs*IVT assay to prioritize primary hits with the potential to specifically inhibit parasite translation.

### Prioritization and assessment of hits.

A total of 7 compounds were identified as attractive primary hits targeting P. falciparum translation. Of these 7, MMV019189 (*Pf*IVT IC_50_, 0.03 μM; *Hs*IVT IC_50_, 7.7 μM), MMV676008 (*Pf*IVT IC_50_, 0.4 μM; *Hs*IVT IC_50_, 12 μM), MMV667494 (*Pf*IVT IC_50_, 0.3 μM; *Hs*IVT IC_50_, >30 μM), and MMV688372 (*Pf*IVT IC_50_, 0.6 μM; *Hs*IVT IC_50_, >30 μM) demonstrated promising potency against parasite translation, with selectivity windows ≥30-fold over inhibition of translation in human lysate (summarized in Table 1). The remaining 3 compounds—MMV634140 (*Pf*IVT IC_50_, 2.9 μM; *Hs*IVT IC_50_, >0 μM), MMV1578897 (*Pf*IVT IC_50_, 0.04 μM; *Hs*IVT IC_50_, 0.18 μM), and MMV1580839 (*Pf*IVT IC_50_, 0.8 μM; *Hs*IVT IC_50_, 8 μM)—also showed significant selectivity in the *Pf*IVT assay versus the *Hs*IVT, albeit with more modest safety windows.

We next assessed the potency of the prioritized compounds against asexual-blood-stage (ABS) P. falciparum (Table 3). With the exception of MMV676008, all 7 of these compounds demonstrated some level of activity against ABS parasites. Three compounds demonstrated submicromolar activity. MMV667494 and MMV634140, both analogues of the *Pf*eEF2 inhibitor M5717/DDD00107498, were the most potent in ABS assay, with 50% effective concentration (EC_50_) values of 10 and 41 nM, respectively. MMV019189 also demonstrated promising activity in ABS assays (EC_50_, 750 nM). In some cases, our prioritized compounds demonstrated higher potency in ABS than in *Pf*IVT assays. Indeed, this was also observed with several established inhibitors of protein translation (Table S1). There are a number of possible explanations for this phenomenon. However, the most likely explanation is that since the potency of competitive inhibitors is dependent on assay conditions, specifically substrate concentrations, the high concentrations of factors such as ATP required to drive the IVT assay may somewhat mask the true potency of inhibitors.

**TABLE 3:** Structures and antimalarial data for compounds demonstrating sub μM activity in the *Pf*IVT assay

Compound (reference)	Structure	P. falciparum asexual-blood-stage EC_50_ (μM)	*Pf*IVT IC_50_ (μM)
MMV019189 (35)	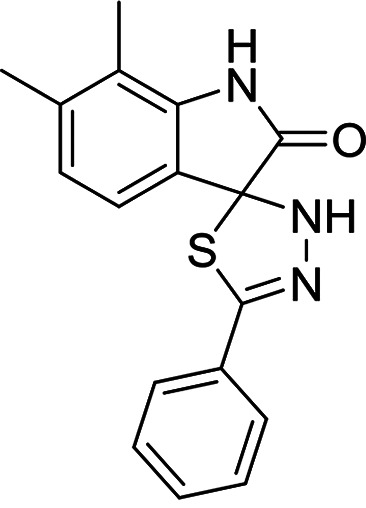	0.75^*a*^	0.03 ± 0.009
MMV1578897 (30)	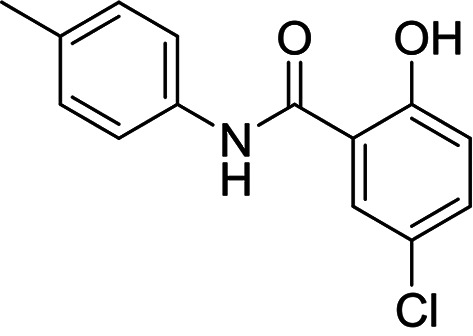	5^*b*^	0.04 ± 0.008
MMV687807 (29)	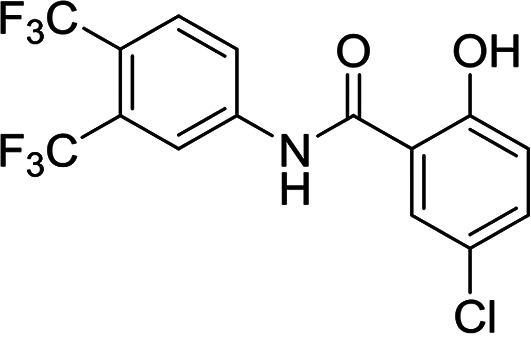	1.8^*a*^	0.09 ± 0.007
MMV667494 (44)	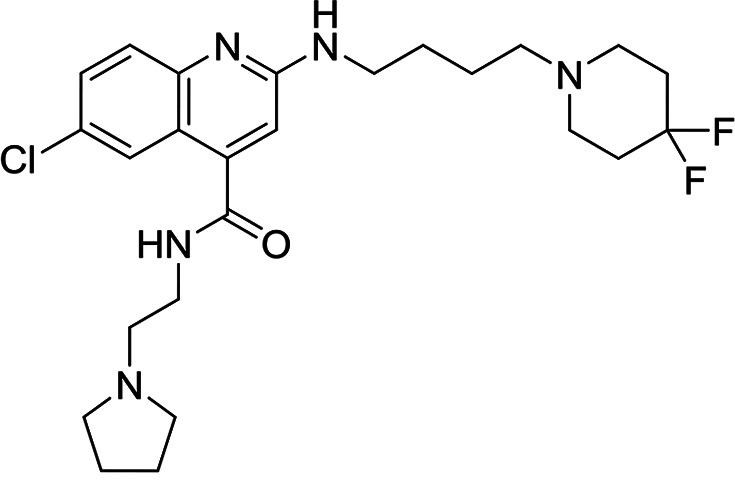	0.010^*a*^	0.3 ± 0.09
MMV676008 (48)	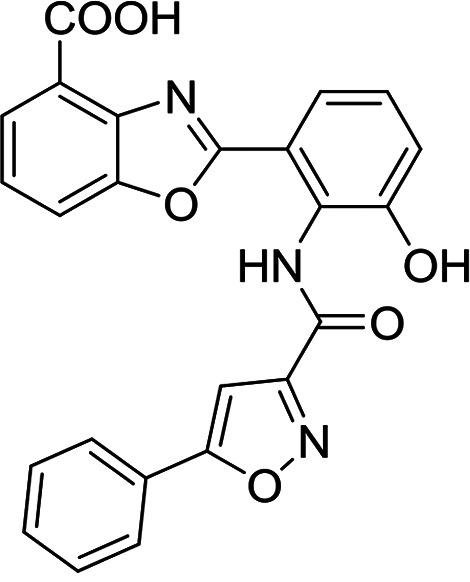	>20^*a*^	0.4 ± 0.08
MMV688372 (31)	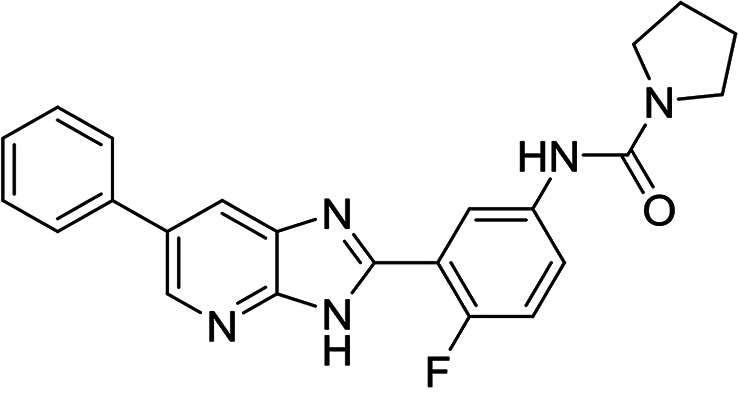	13.6	0.6 ± 0.06
MMV634140 (12)	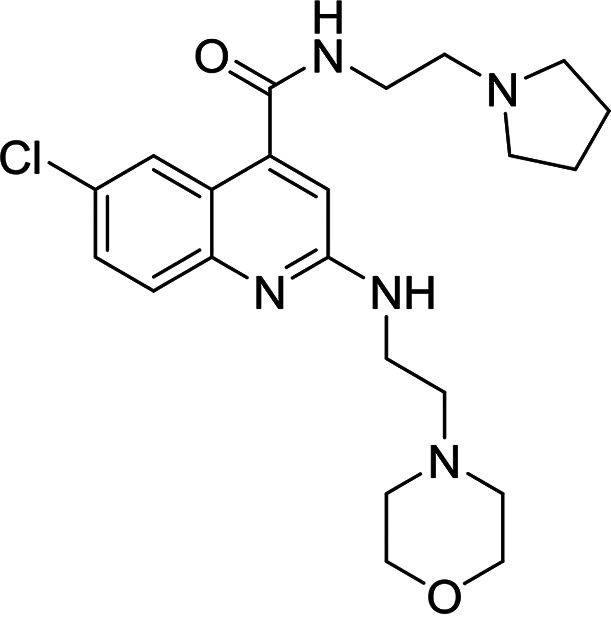	0.09^*a*^	3 ± 0.09
MMV1580839 (34)	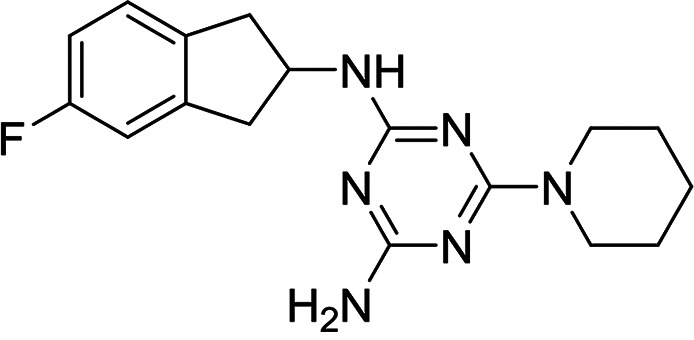	5^*b*^	0.8 ± 0.08

a Data reported for the P. falciparum 3D7 cell line in reference 25.

b Data reported for the P. falciparum Dd2 cell line in reference 49.

MMV687807 is a potent salicylamide inhibitor of asexual blood and gametocyte stages of *Plasmodium* (*Pf*IVT IC_50_, 90 nM, EC_50_, 1.8 μM). However, this likely generic inhibitor of translation was not prioritized due to its potential liability in human cells (*Hs*IVT IC_50_, 500 nM). A second salicylanilide derivative, MMV1578897, also showed submicromolar inhibition of *in vitro* translation (*Pf*IVT IC_50_, 40 nM) but relatively lower activity against blood-stage P. falciparum. For these, MMV1578897 and MMV687807 were considered secondary hits that could potentially be used as a backup series in drug discovery programs targeting translation in *Plasmodium*. It is worth noting that these two compounds share the salicylanilide core structure with the antihelminthic drug niclosamide (28). Both compounds have also been reported as having antitubercular activity and were suspected protonophores (29), with MMV1578897 demonstrating broad activity against a range of bacterial pathogens (30).

Although showing good potency in *Pf*IVT assay, three other hits were classified as lower-priority compounds since they failed to demonstrate concomitant activity/potency against the P. falciparum parasite (Table 3). MMV676008 (*Pf*IVT IC_50_, 40 nM) is a benzoxazole natural product isolated more than 2 decades ago from cultures of *Streptomyces*; however, it showed an EC_50_ value of >25 μM against P. falciparum *in vitro* and thus is not suitable for a hit-to-lead drug discovery program. MMV688372 (*Pf*IVT IC_50_, 600 nM) is an imidazopyridine previously demonstrating low nanomolar activity against T. brucei *in vitro* and *in vivo* (31). MMV688372 also showed modest potency against P. falciparum (EC_50_, 14 μM), though not enough to be prioritized. Interestingly, this compound also shares structural features with two established proteasome inhibitors currently in clinical development for the treatment of visceral leishmaniasis (32, 33). MMV1580839 (*Pf*IVT IC_50_, 900 nM), reported as having an EC_50_ value of 5 μM against P. falciparum, is described as an inhibitor of the bacterial methyltransferases ErmC and ErmAM, blocking the ability of these enzymes to methylate rRNA (34). Finally, the most active confirmed hit in our *Pf*IVT assay, MMV019189 (IC_50_, 30 nM), is a previously reported antimalarial with nanomolar activity against multiple developmental stages of the parasite (35). Undoubtedly, additional work will be required to further define the molecular targets of MMV019189 and other actives described here. One strategy could be to carry out thermal protein profiling (TPP) with compounds using the enriched lysates prepared for our *Pf*IVT assay (36, 37). TPP takes advantage of the fact that binding of a ligand to its target can thermally stabilize the target. This approach enables the thermal stability of all proteins within a lysate to be monitored and compared in the presence and absence of test compounds, thus enabling potential targets to be identified. For compounds where structure-activity relationships are better understood, linkers could be attached and used as handles to facilitate the pulldown of targets from IVT lysates and establish molecular targets. Alternatively, parasites resistant to test compounds can be generated through *in vitro* evolution. Whole-genome analysis of resistant clones can lead to the identification of genomic changes pointing to genes encoding compound targets (38). Our ultimate goal will be to structurally enable drug discovery programs focused on evolving more potent or selective analogues of MMV019189 and other promising hit compounds.

### Conclusions.

In summary, the combination of our IVT assay platform with *in vitro* potency data has led to the identification of one priority hit (MMV019189) and several other compounds of interest that are associated with inhibition of protein synthesis in P. falciparum for the first time. These studies clearly demonstrate the power of this assay platform to generate robust data that can be used to prioritize compounds acting via this highly desirable mechanism of action.

## MATERIALS AND METHODS

### Generation of reporter constructs to support P. falciparum and human IVT assays.

To support *Pf*IVT assays, the production of luciferase mRNA was required. The previously described plasmid pHLH-1, which comprises the 5′ UTR of the P. falciparum histidine-rich protein 3 (*Pf*Hrp3) gene upstream of a firefly luciferase reporter gene and the 3′ UTR from the histidine-rich protein 2 (*Pf*Hrp2) gene downstream (19), was used as a starting point. This plasmid was modified to introduce a T7 bacteriophage terminator sequence using site-directed mutagenesis. Briefly, Phusion DNA polymerase (New England BioLabs [NEB]) was used in PCRs in conjunction with a forward (FW; CGC GCT TGG CGA ATC ATG GTC A) and reverse (RV; GCT AGT TAT TGC TCA GCG GCA ATT AAC CCT CAC TAA AGG GAA CAA AAG) primers under the following conditions: 1× cycle of denaturation at 98°C for 30 s followed by 35 cycles of denaturation at 98°C for 10 s, annealing at 58°C for 15 s, and extension at 72°C for 120 s. The resulting vector (modified pHLH) was linearized with HindIII (NEB), and the PCR-amplified *cbg99* luciferase gene was inserted using Gibson Assembly (NEB). The *cbg99* gene (Fig. S1) was PCR amplified from Promega’s pCBG vector using the Phusion DNA polymerase (NEB) and the following primers: FW, ATA TTA ATA CAG TTA TTT TAA AAA AAT GGT GAA GCG TGA GAA AAA TG, and RV, TTT TAA TCT ATT ATT AAA TAA GCT TCT AAC CGC CGG CC. The resulting plasmid (modified pHLH-CBG99) was used in the production of mRNA for the P. falciparum IVT assay.

To support the *Hs*IVT assay, the pT7CFE vector (Thermo Fisher Scientific), containing an IRES from the encephalomyocarditis virus (EMCV) and a 30-bp poly(A) region, was digested with MscI and XhoI (NEB). The *cbg99* gene, PCR amplified from the pCBG vector using FW primer GAA AAA CAC GAT GAT AAT ATG GCC ACC ATG GTG AAG CGT GAG AAA AAT G and RV primer CAG TGG TGG TGG TGG TGG TGC TCG AGA CCG CCG GC, encompassing homology to pT7CFE. The PCR product was then cloned into the digested pT7CFE vector using Gibson Assembly (NEB). The resulting plasmid (pT7CFE-CBG99) was used in the production of mRNA for the human IVT assay.

### Assay validation construct.

In preliminary assay development experiments, a plasmid that facilitated expression of a hemagglutinin (HA)-tagged version of the firefly luciferase (Fig. S1) was used. In this study, the modified pHLH-1 plasmid, described above, was used as a starting point. The plasmid was digested with NsiI and HindIII (NEB) to release the Cbg luciferase. A Genestring (Life Technologies) containing homology to the digested plasmid was cloned using Gibson Assembly inserting a 3×HA tag and tobacco etch virus (TEV) cleavage site into the vector (Fig. S1). This formed a universal vector (pHLHUNI) that could be used to insert genes of interest fused to an N- or C-terminal 3×HA tag and TEV cleavage site. The newly formed vector was linearized with HindIII and the firefly luciferase (*luc*) gene inserted, again using Gibson Assembly.

### Western blotting.

Proteins were separated on 12% SDS-PAGE gels and then transferred to nitrocellulose membranes using an iBlot 2 (Thermo Fisher) system as per the manufacturer’s instructions. The membrane was blocked in 10% (wt/vol) milk in phosphate-buffered saline (PBS) containing 0.1% Tween 20 (PBS-T) for 1 h. Following blocking, the membrane was incubated in 2% (wt/vol) milk in PBS-T containing the HA tag-specific primary antibody 12CA5 (Cell Signaling) at a dilution of 1:1,000 for 1 h. The membrane was then washed with PBS-T (three times for 5 min). Following a washing, membranes were incubated in 2% milk in PBS-T containing an anti-rabbit secondary antibody conjugated to horseradish peroxidase (HRP; Merck) at a dilution of 1:5,000 for 1 h prior to washing in PBS-T (three times for 5 min). HA-tagged protein on the membrane was detected using the ECL Western blot detection reagent (GE Healthcare) as per the manufacturer’s instructions and visualized using a Gel Doc XR+ imaging system (Bio-Rad).

### *In vitro* transcription of mRNA.

Reactions to produce *cbg99* mRNA were run using 40 mM HEPES (pH 7.4), 18 mM magnesium acetate, ribonucleotide triphosphates (5 mM each), 2 mM spermidine, 40 mM dithiothreitol (DTT), 0.0025 U/μL inorganic pyrophosphatase, modified pHLH-CBG99 for the parasite assay and pT7CFE-CBG99 for the human assay (70 ng/μL), RNase inhibitor (3 U/μL), and T7 RNA polymerase (10 U/μL). This reaction mixture was incubated for 100 min at 37°C, and DNase I (0.1 mg/mL) was added for the final 20 min of the incubation. The reaction mixture was then diluted 1:1 with RNase-free H_2_O and RNA was precipitated using 1.6 M (final concentration) lithium chloride, followed by incubation on ice (30 min) and centrifugation (15,000 × *g*, 20 min, 4°C). The resulting pellet was dissolved in 270 μL RNase-free H_2_O with agitation at 30°C. RNA was precipitated once again by addition of 3 M ammonium acetate (30 μL) and ice-cold absolute ethanol (700 μL). Samples were incubated at −20°C for 30 min, and mRNA was precipitated by centrifugation (15,000 × *g*, 20 min, 4°C). The supernatant was removed and discarded, and the pellet was rinsed twice with 70% ethanol. Residual ethanol was removed, and the pellet was air dried (1 h) prior to solubilization with RNase-free water. The concentration of the resuspended RNA was determined using a NanoDrop spectrophotometer.

### Parasite strain and culture conditions.

The Plasmodium falciparum reference strain 3D7 used throughout this study was cultured as previously described (39). Briefly, cultures incubated at 37°C in a humidified atmosphere of 1% O_2_ and 3% CO_2_ in balance with N_2_ were maintained in RPMI 1640 media supplemented with 5% A^+^ human red blood cells (provided by the Scottish National Blood Transfusion Service), 25 mM HEPES, 2 mM l-glutamine, 0.5% AlbuMAX II (Gibco), 12 mM sodium bicarbonate, 0.2 mM hypoxanthine, and 20 mg/L gentamicin (pH 7.3).

### P. falciparum lysate preparation.

3D7 parasites were synchronized following two rounds of treatment with d-sorbitol (5%), as previously described (40). The hematocrit was reduced from 5% (for standard culture) to 1.5 to 2% and cultures were transferred into HYPERflasks (Corning). Fresh medium was added once or twice daily. Late trophozoite/schizont stage parasites were harvested by centrifugation (1,800 × *g*, 15 min, 4°C, low brake) once parasitemia reached 8 to 15%. Harvested red blood cells (infected) were lysed by incubation in 0.1% (wt/vol) saponin on ice for 10 min with gentle agitation. Free parasites were harvested by centrifugation (2,800 × *g*, 10 min, 4°C) and washed 3 times in wash buffer (WB; 100 mM potassium acetate, 2.5 mM magnesium acetate, 45 mM HEPES [pH 7.4], 250 mM sucrose, 2 mM dithiothreitol, and 15 μM leupeptin) to remove lysed red blood cell debris. The resulting pellet was resuspended in 1 volume of WB supplemented with cOmplete EDTA-free protease inhibitor cocktail (Roche; 1 tablet/20 mL) and human RNase A inhibitor (Sigma; 5 U/mL). Parasite lysis was achieved by nitrogen cavitation using a prechilled 45-mL Parr cell disruption vessel (1,500 lb/in^2^, 60 min, 4°C). To clarify the lysate, it was centrifuged (10,000 × *g*, 15 min, 4°C), and the supernatant was collected, transferred into fresh tubes, and centrifuged (30,000 × *g*, 15 min, 4°C). As a quality assurance step for each lysate, the RNA content of the resulting supernatant was determined using a NanoDrop spectrophotometer (Shimadzu). Supernatants with RNA levels of >250 ng/μL were aliquoted (100 μL), flash frozen in liquid nitrogen, and stored at −80°C.

### HEK 293F cell culture.

FreeStyle human embryonic kidney (HEK) 293F cells (Thermo Fisher Scientific) were grown in 500-mL polycarbonate Erlenmeyer vented flasks (Corning) containing FreeStyle 293 expression medium (Life Technologies) upon reaching a density of approximately 2 × 10^6^. Cells were then centrifuged at 1,000 × *g* for 10 min at 4°C, the medium was discarded, and the resulting pellet was washed once in buffer II containing 20 U of human placental RNase inhibitor (Sigma-Aldrich) and cOmplete EDTA-free inhibitor cocktail (Roche) before being centrifuged at 2,800 × *g* for 10 min. Clarified HEK cell lysate was obtained in the same way as described for P. falciparum lysate.

### P. falciparum and human IVT assays.

*Pf*IVT and *Hs*IVT reactions (5 μL final volume) were performed in 384-well plates (Corning) containing 50% lysate from cells (either P. falciparum or HEK 293F), 10% amino acid solution (final concentration of 400 μM for each amino acid in 60 mM KOH; biotechrabbit), 10% energy recovery solution (final assay concentrations of 40 mM HEPES [pH 7.4], 1.5 mM ATP, 0.15 mM GTP, 40 U/mL creatine phosphate, and 40 U/mL creatine phosphokinase), 10% helper solution (final assay concentrations of 200 μM cystine, 2% polyethylene glycol 3000 [PEG 3000], 1 mM spermidine, 0.5 mM folinic acid, and 15 μM leupeptin), 10% supplemental salt solution (22.5 mM HEPES [pH 7.4], 50 mM potassium acetate, 1 mM magnesium acetate, 1 mM DTT, 1 U/mL human placental RNase inhibitor, 0.1 mM leupeptin), and 10% *cbg99* luciferase mRNA (3,000 ng/mL). A master mix solution containing all components was prepared on ice prior to assays, and 5 μL was dispensed into wells using an automated Integra VIAFLO 16-channel 12.5-μL pipette. Assays were incubated for 210 min at 32°C, and luciferase buffer (5 μL; final assay concentrations of 45 mM HEPES [pH 7.4], 1 mM MgCl_2_, 1 mM ATP, 5 mM DTT, 1% Triton X-100, 10 mg/mL bovine serum albumin [BSA], 1 mg/mL d-luciferin, and 1 × Pierce firefly signal enhancer) was added to read luminescence using a BMG PheraStar plate reader. Cycloheximide was used as a positive control in both *Pf*IVT and *Hs*IVT reactions, representing 100% inhibition. Hits were assessed as inhibitors of translation in both IVT assays in 8-point dose-dependent assays (30 μM to 13.7 nM; 1:3 dilutions). Relative activity of luciferase was calculated based on positive-control reactions.

### False-positive counterscreen.

Hits identified in our primary assay screen were assessed for their potential to interfere with the *cbg99* luciferase reporter. For this counterscreen, the master mix containing all the components required for translation (as described above) was incubated for 210 min at 32°C, resulting in the *in vitro* translation of *cbg99* luciferase. The produced luciferase was then incubated with test compounds for 5 min prior to the addition of luciferase reaction buffer. Luciferase I inhibitor (Calbiochem) was used at 50 μM as a positive control in each assay, representing 100% inhibition. Hits from the primary screen were assessed as luciferase inhibitors in 8-point dose-dependent assays ranging from 30 μM to 13.7 nM (1:3 dilutions). Levels of luciferase inhibition were determined relative to the positive control.

### Inhibitor studies.

Test compounds (10 mM in 100% dimethyl sulfoxide [DMSO]) were dispensed into 384-well assay plates using the acoustic Echo 550 dispenser (Labcyte). Compound libraries were screened at a single inhibitor concentration (30 μM), and compounds inhibiting >50% *Pf*IVT activity were selected for dose-dependent analysis. For dose-response assays, compound concentrations ranging from 30 μM to 13.7 nM (1:3 dilutions) were assessed in 8-point inhibition assays. IC_50_ values were determined using XLFit using a 4-parameter equation. Cycloheximide (50 μM) was used as a positive control for *Pf*IVT on every assay plate representing 100% inhibition.

### Compounds and libraries.

Established inhibitors of IVT—cycloheximide (41), borrelidin (42), halofuginone (11), and emetine (43)—were purchased from Sigma. Open-access compound libraries Pathogen Box and the Pandemic Response Box, both 400-compound libraries, were kindly provided by Medicines for Malaria Venture (MMV). DDD00197451 (44) and DDD01712277 (12) were synthesized as previously described. Cladosporin was kindly provided by Chris Walpole from the Structural Genomics Consortium.

### Quality control of library compounds.

Hits identified in our primary assay screen were subjected to mass spectrometry to confirm purity and compound identity. For this, library compounds in DMSO were diluted 20-fold in a clean, u-shaped, deep-well 384-well plate. The plate was heat sealed (Waters heat sealer) and vortexed. Samples were then injected into an ultrahigh-performance liquid chromatography (UHPLC)-mass spectrometry (MS) 2020 single-quadrupole mass spectrometer with atmospheric pressure chemical ionization and electrospray ionization (ESI) probes fitted (DG-20A3R and DG-20A5R degasser; 2 × LC-30 AD binary pups; SIL-30AC MP multiplate autosampler with sample chiller; CTO-20AC plus 2 column switching valve; SPD-M30A UV/visible diode array detector with 1-cm flow cell). Library compounds (2 μL) were injected into a Hypersil Gold column (Thermo Fisher Scientific; 1.9-μm internal diameter, 50-mm length, 175-Å pore size) and HPLC separated using a gradient of solvent A (water/0.05% formic acid) and solvent B (acetonitrile/0.05% formic acid) at 0.6 mL/min and 50°C. Detection was performed at 254 nm (40°C) using a mass spectral range from 50 to 1,000 Da (both positive and negative modes; scan speed, 15,000 Da/s; interface voltage, 4.5 kV). Shimadzu Labsystems 5.91 software was used to control sample injections into the LC-MS system and to analyze processed data (integration of peak areas and assessment of masses). Trazodone hydrochloride and reserpine were used as internal standards.

### SYBR green-based P. falciparum growth inhibition assay.

Our SYBR green asexual-growth assay was based on a previous report (45). In brief, 96-well black clear-bottom plates (Corning) were preprinted with a compound and normalized with DMSO to 0.5% of a total assay volume of 100 μL. Highly synchronized ring-stage parasites and blood were added to a 2% final parasitemia and 1% hematocrit. The compounds were incubated with parasites for 72 h prior to freezing at −20°C (to aid cell lysis). The plate was thawed on ice and lysis buffer (20 mM Tris pH 7.5, 5 mM EDTA, 0.008% [w/v] saponin, 0.08% [v/v]) containing SYBR green I (Thermo Fisher Scientific) at a final concentration of 0.02% (v/v) was added. The 96-well plate was equilibrated to room temperature for 1 h before fluorescence was determined on a TECAN Infinite Pro 200 microplate reader using green fluorescent protein (GFP) filters (excitation, 485 nm; emission, 535 nm). Data were fitted to a two-parameter equation using GraFit version 7.0 (Erithacus Software), and EC_50_ values were calculated. An excess of the standard inhibitor mefloquine (10 μM) was used to define 0% parasite growth.
