# Supplementary material for: High-Throughput Screening Platform To Identify Inhibitors of Protein Synthesis with Potential for the Treatment of Malaria
Source: Antimicrob Agents Chemother. 2022 Jun 1;66(6):e00237-22. doi: 10.1128/aac.00237-22 (PMC9211397; doi:10.1128/aac.00237-22)
Supplement: Supplemental file 1 — Fig. S1 to S4 and Table S1. Download aac.00237-22-s0001.pdf, PDF file, 0.3 MB [file aac.00237-22-s0001.pdf]

Supplementary information

Firefly luciferase

ATGCATGAAGACGCCAAAAACATAAAGAAAGGCCCGGCGCCATTCTATCCTCTAGAGGATGGAACCGCTGGAGAGCAACTGCATAAGGCTATGAAGAGATACGCCCTGGTTCCT
GGAACAATTGCTTTTACAGATGTACATATCGAGGTGAACATCACGTACGCGGAATACTTCGAAATGTCCGTTCGGTTGGCAGAAGCTATGAAACGATATGGGCTGAATACAAATC
ACAGAATCGTCGTTTGCAGTGAAAACTCTCTTCAATTCTTTATGCCGGTGTTGGGCGCGTTATTTATCGGAGTTGCAGTTGCGCCCGCGAACGACATTTATAATGAACGTGAATTG
CTCAACAGTATGAACATTTCGCAGCCTACCGTAGTGTTTGTTTCCAAAAAGGGGTTGCAAAAAATTTTGAACGTGCAAAAAAAATTACCAATAATCCAGAAAATTATTATCATGGAT
TCTAAAACGGATTACCAGGGATTTCAGTCGATGTACACGTTCGTCACATCTCATCTACCTCCCGGTTTTAATGAATACGATTTTGTACCAGAGTCCTTTGATCGTGACAAAACAATT
GCACTGATAATGAATTCCTCTGGATCTACTGGGTTACCTAAGGGTGTGGCCCTTCCGCATAGAACTGCCTGCGTCAGATTCTCGCATGCCAGAGATCCTATTTTTGGCAATCAAA
TCATTCCGGATACTGCGATTTTAAGTGTTGTTCCATTCCATCACGGTTTTGGAATGTTTACTACACTCGGATATTTGATATGTGGATTTCGAGTCGTCTTAATGTATAGATTTGAAG
AAGAGCTGTTTTTACGATCCCTTCAGGATTACAAAATTCAAAGTGCGTTGCTAGTACCAACCCTATTTTCATTCTTCGCCAAAAGCACTCTGATTGACAAATACGATTTATCTAATTT
ACACGAAATTGCTTCTGGGGGCGCACCTCTTTCGAAAGAAGTCGGGGAAGCGGTTGCAAAACGCTTCCATCTTCCAGGGATACGACAAGGATATGGGCTCACTGAGACTACATC
AGCTATTCTGATTACACCCGAGGGGGATGATAAACCGGGCGCGGTCGGTAAAGTTGTTCCATTTTTTGAAGCGAAGGTTGTGGATCTGGATACCGGGAAAACGCTGGGCGTTAA
TCAGAGAGGCGAATTATGTGTCAGAGGACCTATGATTATGTCCGGTTATGTAAACAATCCGGAAGCGACCAACGCCTTGATTGACAAGGATGGATGGCTACATTCTGGAGACATA
GCTTACTGGGACGAAGACGAACACTTCTTCATAGTTGACCGCTTGAAGTCTTTAATTAAATACAAAGGATATCAGGTGGCCCCCGCTGAATTGGAATCGATATTGTTACAACACCC
CAACATCTTCGACGCGGGCGTGGCAGGTCTTCCCGACGATGACGCCGGTGAACTTCCCGCCGCCGTTGTTGTTTTGGAGCACGGAAAGACGATGACGGAAAAAGAGATCGTGG
ATTACGTCGCCAGTCAAGTAACAACCGCGAAAAAGTTGCGCGGAGGAGTTGTGTTTGTGGACGAAGTACCGAAAGGTCTTACCGGAAAACTCGACGCAAGAAAAATCAGAGAGA
TCCTCATAAAGGCCAAGAAGGGCGGAAAGTCCAAATTGTAA

Click beetle luciferase (*cbg99*)

ATGGTGAAGCGTGAGAAAAATGTCATCTATGGCCCTGAGCCTCTCCATCCTTTGGAGGATTTGACTGCCGGCGAAATGCTGTTTCGTGCTCTCCGCAAGCACTCTCATTTGCCTC
AAGCCTTGGTCGATGTGGTCGGCGATGAATCTTTGAGCTACAAGGAGTTTTTTGAGGCAACCGTCTTGCTGGCTCAGTCCCTCCACAATTGTGGCTACAAGATGAACGACGTCG
TTAGTATCTGTGCTGAAAACAATACCCGTTTCTTCATTCCAGTCATCGCCGCATGGTATATCGGTATGATCGTGGCTCCAGTCAACGAGAGCTACATTCCCGACGAACTGTGTAAA
GTCATGGGTATCTCTAAGCCACAGATTGTCTTCACCACTAAGAATATTCTGAACAAAGTCCTGGAAGTCCAAAGCCGCACCAACTTTATTAAGCGTATCATCATCTTGGACACTGT
GGAGAATATTCACGGTTGCGAATCTTTGCCTAATTTCATCTCTCGCTATTCAGACGGCAACATCGCAAACTTTAAACCACTCCACTTCGACCCTGTGGAACAAGTTGCAGCCATTC
TGTGTAGCAGCGGTACTACTGGACTCCCAAAGGGAGTCATGCAGACCCATCAAAACATTTGCGTGCGTCTGATCCATGCTCTCGATCCACGCGTGGGCACTCAGCTGATTCCTG
GTGTCACCGTCTTGGTCTACTTGCCTTTCTTCCATGCTTTCGGCTTTAGCATTACTTTGGGTTACTTTATGGTCGGTCTCCGCGTGATTATGTTCCGCCGTTTTGATCAGGAGGCT
TTCTTGAAAGCCATCCAAGATTATGAAGTCCGCAGTGTCATCAACGTGCCTAGCGTGATCCTGTTTTTGTCTAAGAGCCCACTCGTGGACAAGTACGACTTGTCTTCACTGCGTG
AATTGTGTTGCGGTGCCGCTCCACTGGCTAAGGAGGTCGCTGAAGTGGCCGCCAAACGCTTGAATCTTCCAGGGATTCGTTGTGGCTTCGGCCTCACCGAATCTACCAGCGCT
AACATTCACTCTCTCGGGGATGAGTTTAAGAGCGGCTCTTTGGGCCGTGTCACTCCACTCATGGCTGCTAAGATCGCTGATCGCGAAACTGGTAAGGCTTTGGGCCCGAACCAA
GTGGGCGAGCTGTGTATCAAAGGCCCTATGGTGAGCAAGGGTTATGTCAATAACGTTGAAGCTACCAAGGAGGCCATCGACGACGACGGCTGGTTGCATTCTGGTGATTTTGGA
TATTACGACGAAGATGAGCATTTTTACGTCGTGGATCGTTACAAGGAGCTGATCAAATACAAGGGTAGCCAGGTTGCTCCAGCTGAGTTGGAGGAGATTCTGTTGAAAAATCCAT
GCATTCGCGATGTCGCTGTGGTCGGCATTCCTGATCTGGAGGCCGGCGAACTGCCTTCTGCTTTCGTTGTCAAGCAGCCTGGTAAAGAAATTACCGCCAAAGAAGTGTATGATT
ACCTGGCTGAACGTGTGAGCCATACTAAGTACTTGCGTGGCGGCGTGCGTTTTGTTGACTCCATCCCTCGTAACGTAACAGGCAAAATTACCCGCAAGGAGCTGTTGAAACAATT
GTTGGAGAAGGCCGGCGGTTAG

Genestring (3× HA)

GTTATTTTAAAAAAATGCATTATCCTTATGACGTACCTGATTATGCTTACCCTTACGATGTGCCAGATTACGCATATCCTTATGATGTGCCTGATTATGCAGACTACGACATACCA
ACAACTGAAAACCTTTACTTTCAGGGCAAGCTTATTTAATAATAGATTAAA

Figure S1. Reporter construct generation. Sequences of the Firefly luciferase (A) and CBG99 luciferase (B) genes. (C) Genestring used to introduce a 3× HA tag and TEV cleavage site into the **modified pHLH-1** reporter plasmid.

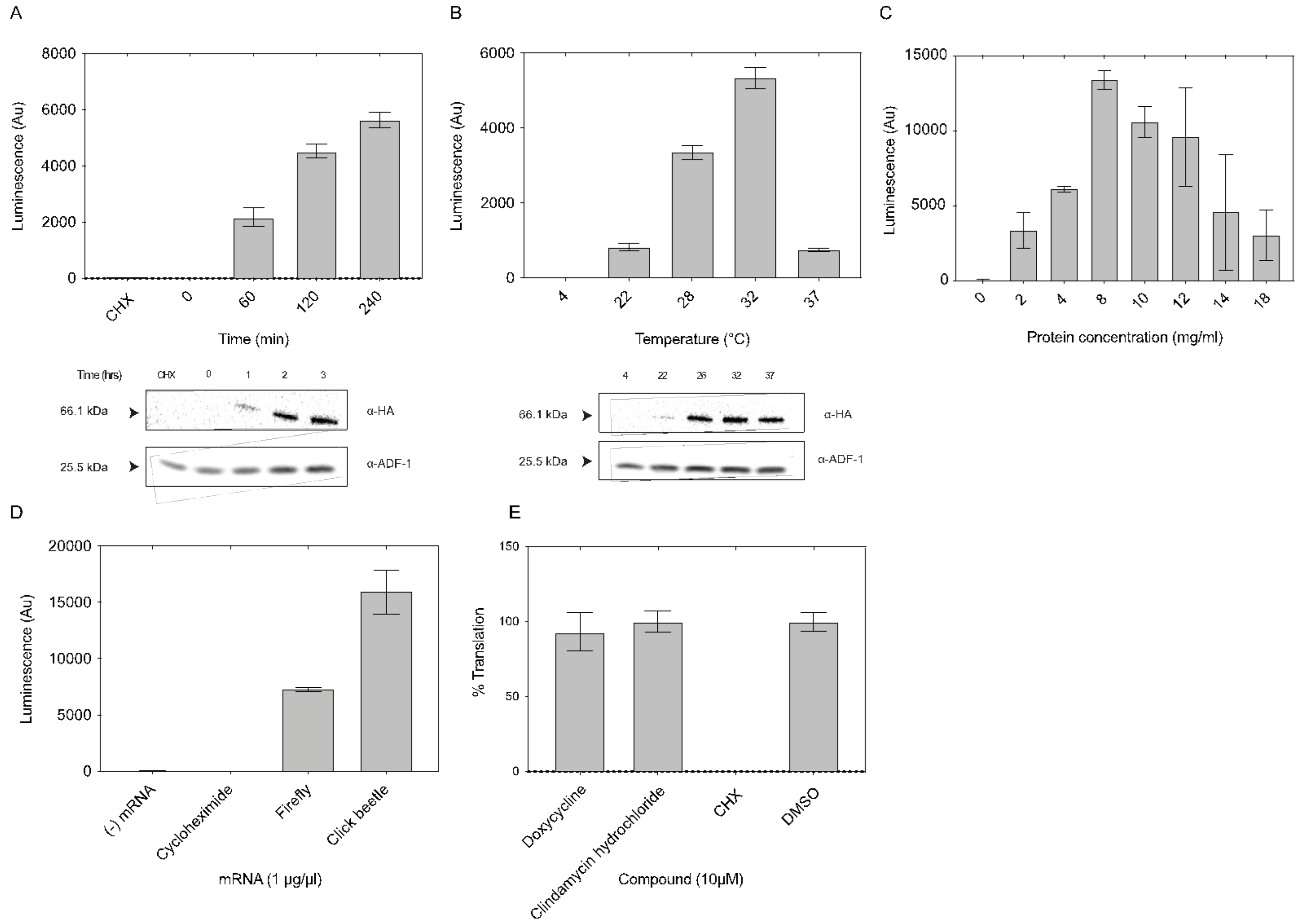

Figure S2. Optimisation of the *Pf*IVT assay. Unless otherwise stated, 1 µg/µL CBG mRNA was used in each reaction alongside 12 mg/mL lysate with a reaction time of 2 h and 30 min at 32 °C. (A) The effect of increasing the assay time on the translation of the luciferase reporter is shown on the graph. Western blots showing the correlation between luminescence signal intensity and HA-tagged protein production is shown below.

(B) The effect of increasing assay temperature on luciferase production. Western blots showing the correlation between luminescence signal intensity and HA-tagged protein production is shown below. (C) The effect of varying lysate concentration on luciferase translation. (D) Direct comparison of firefly luciferase and click beetle (CBG99) luciferase added to each reaction with cycloheximide (CHX) used as a negative control (10 μM). (E) Monitoring the effect of specific inhibitors of apicoplast translation (doxycycline and clindamycin hydrochloride) on the IVT assay. CHX was once again used as a negative control compared to cycloheximide. All data represents the mean ± SD of three technical replicates.

A

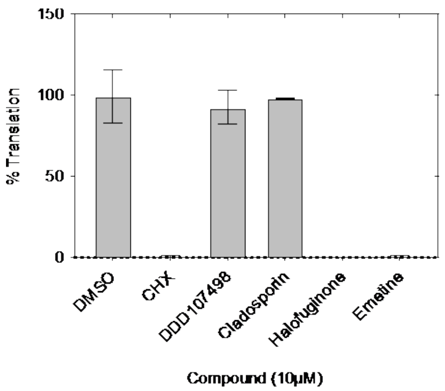

B

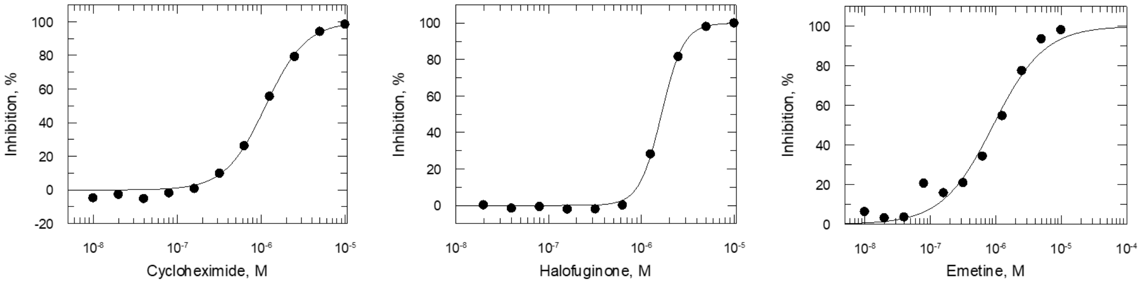

Figure S3. Characterisation of the *Hs*IVT assay. (A) Monitoring the effect of known inhibitors of translation on the *Hs*IVT assay. Specific inhibitors of *P. falciparum* translation DDD0107498 and cladosporin had little or no effect on translation in this assay. However, generic inhibitors of protein translation (CHX, halofuginone and emetine) ablated activity in the assay at 10 µM. Data represents the mean ± SD of three technical replicates. (B) IC_{50} values for cycloheximide, emetine and halofuginone in the *Hs*IVT assay were 1.2 ± 0.04, 0.8 ± 0.1 and 1.7 ± 0.04 µM, respectively. All curves are from a single technical replicate and are representative of data in two biological replicates. IC_{50} values are weighted mean ± SD from at least two biological replicates.

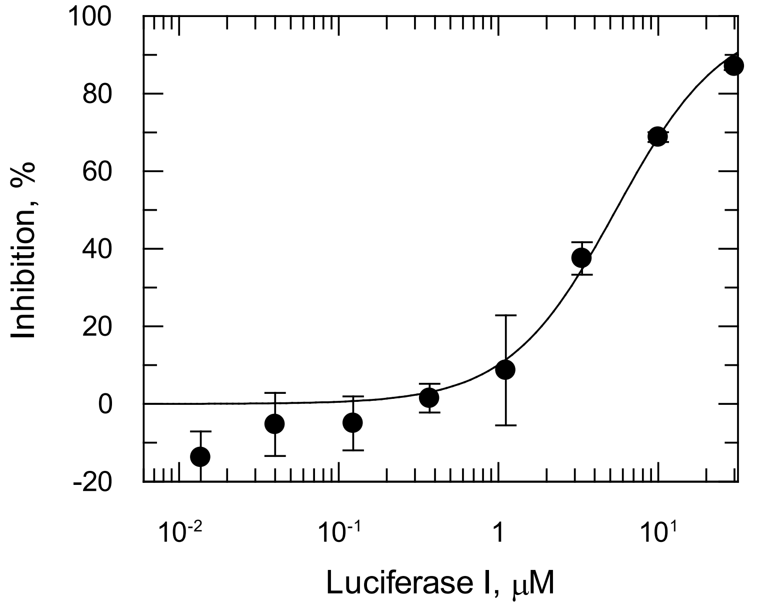

Figure S4. Validation of a CBG99 luciferase-specific counter-screen using the established inhibitor Luciferase-I. This specific inhibitor returned an IC_{50} value of 5.5 ± 0.8 μM. Data is the mean ± SD of three technical replicates.

Table S1: Collated *Pf*IVT and ABS assay data for established inhibitors of protein translation.

Compound	Molecular target	IC_{50} / EC_{50} values, μM	
		*Pf*IVT	*Pf*ABS
Cycloheximide	60S ribosomal subunit (48)	0.5	0.1
Borrelidin	threonyl-tRNA synthetase (50)	1.2	0.002 (50)
Halofuginone	prolyl-tRNA synthetase (51)	0.3	0.0004
Emetine	40S ribosomal subunit (20)	1.8	0.03
DDD01712277	lysyl-tRNA synthetase (6)	0.2	0.1
Cladosporin	lysyl-tRNA synthetase (6)	13	0.07
DDD107498/M5717	translation elongation factor 2 (10)	0.1	0.0008
DDD00197451	translation elongation factor 2 (10)	0.5	0.003
